# Reorganization of brain connectivity across the spectrum of clinical cognitive decline

**DOI:** 10.1007/s10072-024-07688-1

**Published:** 2024-07-30

**Authors:** Demet Yüksel Dal, Zerrin Yıldırım, Hakan Gürvit, Alkan Kabakçıoğlu, Burak Acar

**Affiliations:** 1 Department of Electrical & Electronics Engineering, Boğaziçi University, 34342 İstanbul, Turkey; 2 Department of Neurology, Bağılar Training and Research Hospital, 34212 İstanbul, Turkey; 3 Department of Neurology, Faculty of Medicine, İstanbul University, 34093 İstanbul, Turkey; 4 Neuroimaging Unit, Hulusi Behçet Life Sciences Research Lab, İstanbul University, 34093 İstanbul, Turkey; 5 Department of Physics, Koç University, 34450 İstanbul, Turkey

**Keywords:** Brain connectome, Brain structure and function, Dementia, Alzheimer’s disease, Assortativity

## Abstract

Clinical cognitive decline, leading to Alzheimer’s Disease Dementia (ADD), has long been interpreted as a disconnection syndrome, hindering the information flow capacity of the brain, hence leading to the well-known symptoms of ADD. The structural and functional brain connectome analyses play a central role in studies of brain from this perspective. However, most current research implicitly assumes that the changes accompanying the progression of cognitive decline are monotonous in time, whether measured across the entire brain or in fixed cortical regions. We investigate the structural and functional connectivity-wise reorganization of the brain without such assumptions across the entire spectrum. We utilize nodal assortativity as a local topological measure of connectivity and follow a data-centric approach to identify and verify relevant local regions, as well as to understand the nature of underlying reorganization. The analysis of our preliminary experimental data points to statistically significant, hyper and hypo-assortativity regions that depend on the disease’s stage, and differ for structural and functional connectomes. Our results suggest a new perspective into the dynamic, potentially a mix of degenerative and compensatory, topological alterations that occur in the brain as cognitive decline progresses.

## Introduction

Alzheimer’s Disease Dementia (ADD) accounts for $60-80\%$ of all cases of dementia, causing immense economic and social burden [1, 2]. Though the exact cause and underlying processes are unknown, ADD is commonly accepted as the result of neurodegeneration, characterized by the gradual degradation of brain cells and loss of neurons [3]. This neurodegeneration is thought to have adverse effects on structural connections between brain regions, potentially leading to observed degradation in functional associations/connections, which aligns with the concept of disconnectivity syndrome. Evidence reported in the literature suggests that ADD is a type of disconnectivity syndrome [4, 5]. Hence, studying the structural and functional connections of the brain, a.k.a. connectomics, has attracted attention in the research community.

Structural and functional brain network models (brain connectomes) were proposed and are being used to systematically study the brain organization [6]. Brain connectomes are grouped as structural (sNET) and functional (fNET) models, corresponding to the structural connections between predetermined cortical regions and their functional associations, respectively. The graph theory has been widely utilized as a well-established tool to study these network models (graphs), as it offers local and global measures representing different aspects of connectomes.

The majority of connectomics research on ADD focused on functional connectomes (fNETs). Primarily, statistical assessments of the clustering coefficient, the path length, and the small-worldness, which is a combination of the two measures, of fNETs, between healthy individuals and ADD cases were studied. Statistically significant changes in fNET small-worldness, indicating a disruption in efficiency in communication, have been repeatedly reported for ADDs compared to control groups [7–10]. Supekar et al. used wavelet correlation as the functional connectivity and observed a significant decrease in the global clustering coefficient and no change in the average path length of ADDs compared to the control group [7]. Sanz et al. observed no significant change in the global clustering coefficient and a decrease in average path length using synchronization likelihood as functional connectivity, while Zhao et al. and Liu et al. observed a higher global clustering coefficient and average path length for ADDs compared to control groups using Pearson and partial correlation, respectively [8–10]. Xiang et al. reported a decrease in the global clustering coefficient and an increase in path length using partial correlation, and Li et al. reported a decrease in the global clustering coefficient using Pearson correlation in ADDs compared to control groups [11, 12]. These studies used the Automated Anatomical Labeling (AAL) atlas [13].

The structural connectomes are much less studied and the associated literature is scarce. Coninck et al. reported a higher average path length in sNETs for ADD cases compared to the control group using AAL atlas [14]. Fischer et al. and Lo et al. revealed an unchanged clustering coefficient and a higher average path length in sNETs for ADD cases compared to the control group using Harvard-0xford^1^ and AAL atlas, respectively [15, 16]. Pereira et al. and Wang et al. reported a higher clustering coefficient and average path length in sNETs for ADD cases compared to the control group using Desikan atlas and AAL atlas [17, 18].

Limited work in literature proposed diagnostic classifiers that were trained on selected local connectome features. These studies, largely suffer from the *curse of dimensionality*, which is a problem arising due to using very high dimensional feature spaces with small datasets, hence a sparse coverage of these high dimensional feature spaces. Schouten et al. computed nodal strength, degree, clustering coefficient, and betweenness-centrality for all 110 nodes of the sNET they had built, to discriminate 77 ADDs and 173 controls [19]. Ebadi et al. employed 11 local sNET features computed for the 41 Brodmann areas to classify a cohort of 15 ADD, 15 MCI, and 15 controls (HC) [20]. They reported optimal performance when 430 features were used for AD-MCI and AD-HC classifications and 110 features for the MCI-HC classification. Khazaee et al. used global and local graph measures of fNETs, such as global/local efficiency and betweenness centrality from fNET, to discriminate 20 ADs from 20 controls [21]. They achieved the best performance diagnostic performance using a set of 21 local and global features.

These prior research results in the literature strongly suggest the existence of structural and functional organizational changes in the human brain across the spectrum of dementia, that are captured by fNETs and to a lesser degree by sNETs, but fail to present a coherent and consistent picture due to conflicting findings. This may be primarily due to focusing on global changes and/or overlooking potential spatial variability as well as the variations in the nature of local reorganization across the spectrum of the cognitive condition. Hence, in this preliminary study, we investigated the local structural and functional changes in brain connectivity at different stages of clinical cognitive decline without making any assumption about the type or spatial stationarity of the change. Our findings suggest a localized reorganization that varies in space and character, and also differs for structure and function, at successive stages of cognitive decline. Such an approach to studying brain connectomes not only has the potential to offer a low-dimensional (hence robust) diagnostic biomarker set but also a new perspective to develop insights into the brain’s reorganization that may hint at the underlying degenerative and compensatory mechanisms.

**Fig. 1:**
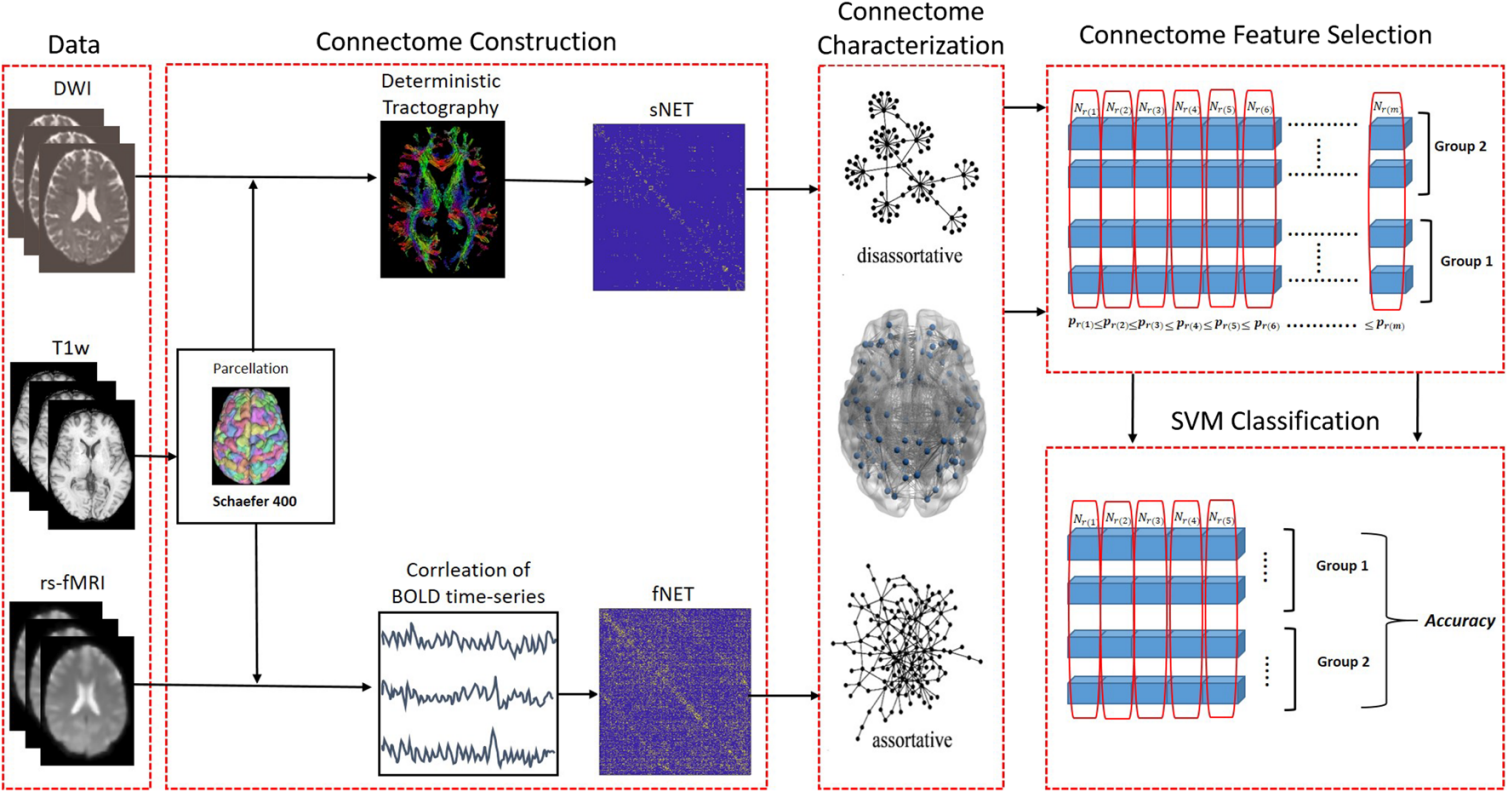
The analysis pipeline is composed of data collection, connectome construction, characterization, feature (nodal assortativity) computation, feature selection, and classifier training/testing. $r_{(i)}$ refers to the node ID with importance ranking *i* and $p_{r_{(i)}}$ is the corresponding p-value computed by t-test

## Materials and methods

Our end-to-end analysis pipeline is summarized in Fig. 1. The main blocks are data acquisition, brain connectome construction, connectome characterization, connectome feature selection, and connectome classification.

### Data acquisition

T1 weighted MRI data was acquired using a 3D FFE (Fast Field Echo) pulse sequence with multi-shot TFE (Turbo Field Echo) imaging mode using the Philips Achieva 3T MRI system (Netherlands) with a 32-channel head coil at Istanbul University, Istanbul Faculty of Medicine, Neuroimaging Unit of Hulusi Behçet Life Sciences Research Laboratory. The acquisition parameters were $TE/TR=3.8\,ms /8.3\,ms, flip\,angle=8^\circ , FOV = 220 \times 240\,mm^2$, $1.0\,mm^3$ isotropic voxels and 180 slices. Diffusion-weighted imaging (DWI) data was acquired with 120 gradient directions with a maximum gradient strength of $40\,mT/m$ and a high slew rate of $200\,mT/m/ms$, at 6 shells in q-space (b-values ranging in $0 - 3000 s/mm^2$). FOV was $220 \times 240\,mm^2$ with imaging matrix $112 \times 112$ and 71 slices with slice thickness of $2.27\,mm$. rs-fMRI data is acquired using the Fast Field Echo (FFE) technique in a multi-slice mode with a single-shot EPI sequence. The acquisition parameter were $TE/TR=30\,ms / 3000\,ms, flip\,angle=80^\circ , FOV = 212\times 194\,mm^2$. 200 EPI volumes and 48 axial slices were acquired with eyes-closed.

### Cohort and clinical assessment

The study cohort is composed of 88 participants signed up following a written consent in accordance with the ethical committee approval^2^. Individuals with a clinical cognitive impairment diagnosis at any stage based on NIA-AA criteria or clinical cognitive tests and their relatives without any cognitive impairment (CI) symptoms, individuals with no CI symptoms and no relatives with any CI diagnosis, provided that they had not started (or changed the dosage of) neurological medication within the last 3 months are included in the study. The individuals with a history of current or past neurological or psychiatric disorders adversely affecting cognition, alcohol or substance abuse, major head trauma with loss of consciousness, and those who had white matter hyperintensities on MRI with a Fazekas score of 2 and 3, were excluded from the study. Contraindications for scanning at MRI were another exclusion criterion from our study.

All participants were clinically assessed following the NIA-AA criteria and grouped into early-stage Alzheimer’s Disease Dementia (ADD) (n = 18), mild cognitive impairment of the amnestic type (MCI) (n = 46), and subjective cognitive impairment (SCI) (n = 24) [22]. The clinical diagnosis of amnestic type MCI is based on identifying an objective deficit in episodic memory with a total free recall score (TFR) of 27 or lower and a Cue Index (CI) of 0.67 or lower on the Free and Cued Selective Reminding Test (FCSRT) [23]. Participants scoring above 27 in TFR-FCSRT or above 0.67 in CI-FCSRT were classified as SCI. The chosen TFR cutoff of 27 was more liberal than 24, a highly sensitive predictor of future dementia in dementia-free individuals [24]. ADD participants met NIA-AA criteria for probable AD with an amnestic presentation [25]. SCI subjects were project volunteers responding to advertisements, scoring at least 1 on either the Cognitive Functions Instrument - Subject form (CFI-S) or CFI - Study Partner form (CFI-SP) [26]. Comprehensive neurological and neuropsychological examinations, along with cranial magnetic resonance imaging (MRI), confirmed SCI, MCI, and ADD diagnoses by a panel of behavioral neurologists. The panel ensured that ADD participants had a Clinical Dementia Rating (CDR) score of 0.5 or 1, indicating very mild or mild dementia; MCI patients had a CDR score of 0.5 and CDR-Sum of Boxes (CDR-SOB) score of 0.5 or 1; and SCI subjects had a CDR score of 0. As such, SCI group serves as the control group within the scope of this study. Table 1 provides a summary of the study cohort.

**Table 1:** Summary of the cohort of participants

	SCI (n=24)	MCI (n=46)	ADD (n=18)
Age	$56.95 \pm 7.54$	$62.04 \pm 9.78$	$69.83 \pm 8.52$
Gender (F/M)	12/12	23/23	8/10
CI-FCRST	$0.88 \pm 0.10$	$0.68 \pm 0.15$	$0.36 \pm 0.21$
TFR-FCRST	$33.45 \pm 3.85$	$20.63 \pm 5.46$	$6.33 \pm 7.23$

### Brain connectome construction

T1 weighted MRI, fMRI, and DWI data are preprocessed and co-registered at 1.5*mm* isotropic resolution, using an in-house preprocessing pipeline^3^. Our pipeline utilizes open-source FreeSurfer, FSL, and Tortoise toolboxes. Co-registered volumes are manually confirmed by neurologists.

Diffusion tensor images (DTI) were reconstructed^4^ from multi-shell (b-values: $0 - 3000 s/mm^2$) DWI data using the dual tensor basis solution to the Stejskal-Tanner equations [27]. We used the 4th order Runge-Kutta (RK4) method for deterministic tractography with 0.15 minimum FA (Fractional anisotropy), 0.7*mm* minimum stepsize, 20*mm* minimum fiber length and $35^\circ$ maximum angle as a curvature threshold [28]. All white-matter voxels with $FA\le 0.15$ were seeded 30 times, initiating bi-directional tractography. sNET edge weights between two nodes (cortical parcels) are determined by the total number of fibers.

A single summary BOLD signal per cortical parcel is computed by using the principal left singular vector of the matrix of BOLD signals of all voxels within a parcel as its reference signal [29]. The partial correlation coefficient between the summary BOLD signals are used as fNET edge weights [30]. The fNETs, thus constructed, are fully connected networks. They are sparsified and regularized using elastic-net regression which is a linear combination of L1-norm and L2-norm regularization [31].

Undirected sNETs and fNETs are represented as $\mathcal {G}^{s/f}=\{{N}^{s/f}, {E}^{s/f} ,\mathbf {{A}}^{s/f}\}$, respectively. *N* and *E* are sets of nodes and edges, respectively, while $\textbf{A}$ is a square and symmetric adjacency matrix that carries the weights of the edges. Nodes correspond to cortical regions defined by a given brain atlas that partitions the cortex into *N* non-overlapping volumes. We used the Schaefer atlas with 400 nodes to build sNETs and fNETs [32].

### Global and nodal (local) network assortativity

The assortativity coefficient ($r\in [-1,1]$) is defined as the correlation coefficient between degrees of neighboring (connected) nodes throughout a network. As such, it is a measure of degree-wise similarity of connected nodes, formally defined as [33],

1 $$\begin{aligned} r = \frac{\sum _{i j}(d_i d_j / N)-(\sum _{i j}(d_i + d_j / 2N))^2}{\sum _{i j}(d_i^2 + d_j^2 )/ 2N - (\sum _{i j}(d_i + d_j / 2N))^2 } \end{aligned}$$

where double sums are over pairs of connected nodes, *N* is the total number of nodes in the network and $d_i$ is the degree of $i^{th}$ node which is essentially the number of neighbors in a binary network. Networks with positive and negative assortativity coefficients are called assortative and disassortative networks, respectively. Assortative networks are more robust to edge removal, hence increasing assortativity coefficient is related to improved network resilience. In disassortative networks, there is a structural tendency for high-degree nodes to act as hubs that connect low-degree nodes. This increases network vulnerability.

Consistent findings from previous research have revealed that brain networks exhibit both high topological efficiency and robustness, along with a tendency to minimize wiring costs [34]. The connectome of the brain is a product of an economical trade-off between minimizing the physical connection cost and maximizing the topological value. The brain can efficiently process information and support complex adaptive behavior by incorporating both cost-efficient and strategically important long-distance connections. Any disturbance to this delicate balance can significantly affect brain health and functioning.

**Table 2:** The accuracies of linear SVM classifiers computed over leave-one-out experiments for SCI-vs-MCI, MCI-vs-ADD, and SCI-vs-ADD tasks on sNETs and fNETs, using 5*D* feature vectors composed of a particular local characteristic (Nodal Assortativity / NA, Clustering Coefficient / CC, Betweenness Centrality / BC, Node Strength / NS, Node Degree / ND) of the statistically most significant nodes (selected for each feature separately), and using 400-D feature vectors (*) composed of the chosen local characteristic of all nodes (full connectome)

	Task	NA	NA$^\dagger$	CC	BC	NS	ND
sNET	SCI-MCI	0.75 / 0.54*	0.50 / 0.49*	0.77 / 0.60*	0.73 / 0.55*	0.74 / 0.53*	0.70 / 0.56*
	MCI-ADD	0.80 / 0.50*	0.50 / 0.48*	0.66 / 0.53*	0.74 / 0.60*	0.66 / 0.55*	0.72 / 0.64*
	SCI-ADD	0.85 / 0.56*	0.49 / 0.48*	0.75 / 0.52*	0.82 / 0.52*	0.75 / 0.68*	0.78 / 0.75*
fNET	SCI-MCI	0.81 / 0.52*	0.50 / 0.49*	0.78 / 0.48*	0.78 / 0.58*	0.70 / 0.50*	0.68 / 0.53*
	MCI-ADD	0.75 / 0.46*	0.49 / 0.48*	0.65 / 0.62*	0.66 / 0.43*	0.76 / 0.57*	0.70 / 0.52*
	SCI-ADD	0.90 / 0.57*	0.49 / 0.48*	0.72 / 0.65*	0.78 / 0.45*	0.77 / 0.50*	0.72 / 0.53*

$^\dagger$ The mean accuracy of random shuffling experiments

Murakami et al. investigated the relation between global assortativity and the robustness and efficiency of networks [35]. They concluded that an increase in assortativity correlates with high average hop count and low efficiency for information diffusion, higher robustness against failures of high-degree nodes, lower robustness against random node failures and concentration of communication loads on a few connections.

The global assortativity coefficient (*r*) fails to capture the local topological variations within a network. To circumvent this limitation, local (nodal) assortativity coefficient ($r_i\in [-1,1]$) is defined as [36],

2 $$\begin{aligned} \delta _{i}&=\frac{1}{d_{i}}\sum _{j\in \textbf{N}_i} | d_{j} - d_{i} |\end{aligned}$$

3 $$\begin{aligned} \bar{\delta }_{i}&=\frac{\delta _i}{\sum _{j=1}^m \delta _j}\end{aligned}$$

4 $$\begin{aligned} r_{i}&= \frac{r+1}{m}-\bar{\delta }_{i} \end{aligned}$$

where $\delta _{i}$ is the mean difference between the degree of a node and those of its neighbours, *m* is the number of nodes in the network. Nodal assortativity coefficients of all 400 nodes, as defined by the Schaefer atlas, were computed for both sNETs and fNETs using the Brain Connectivity Toolbox [37].

### Identification and verification of relevant local changes

In order to localize the significant topological changes, as represented by the local assortativity, across the clinical cognitive decline spectrum, we have posed two discrimination tasks that cover different stages of the spectrum, SCI-vs-MCI, and MCI-vs-ADD. We proposed to use a 2-stage approach to identify and verify the local regions that undergo significant structural and/or functional organization. To this end, we first applied null hypothesis testing (two-sample t-test) to each individual nodal assortativity for SCI-vs-MCI and MCI-vs-ADD, separately, with the null hypothesis being the samples observed come from normal distributions with equal means and unequal and unknown variances. All nodes, with a p-value $\le$ 0.05, are ranked with increasing p-values for both tasks separately. The top 5 nodes are used to train and test binary classifiers for the aforementioned tasks using leave-one-out (LOO) cross-validation approach. The dimension is fixed at 5, which offers a good compromise to achieve robustness and avoid the “curse of dimensionality”. The classifier performances serve as verification of the significance of the identified regions that undergo organizational changes. A qualitative neurological assessment of the findings is also provided by expert neurologists.

As a further confirmation of statistical significance, we randomly shuffled SCI/MCI/ADD labels of all subjects and repeated the node-ranking process described above. More specifically, we assessed the differences in nodal assortativities for SCI-vs-MCI and MCI-vs-ADD, separately for each node, using the randomly assigned labels to subjects and two-sample t-test. The most significant top 5 nodes, thus identified, were used to train and test linear SVM classifiers for the shuffled SCI/MCI/ADD labels. The results are reported in Table 2, together with the results obtained using the true labels.

## Results

### Global and nodal assortativity across the clinical cognitive decline spectrum

**Fig. 2:**
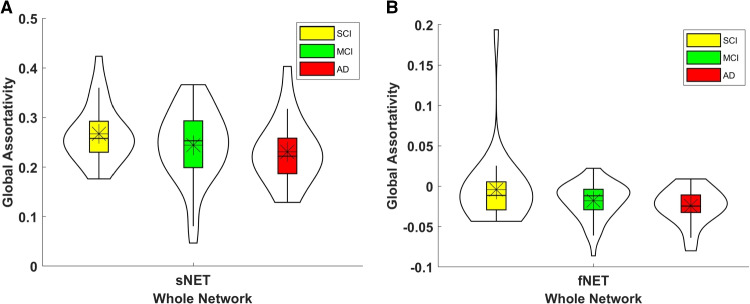
Distributions of global assortativity coefficients (*r*) of **(A)** sNETs, and **(B)** fNETs in the ADD, MCI and SCI groups. sNETs show assortative structure, while fNETs are primarily disassortative. There is no statistically significant difference between different stages of clinical cognitive decline (p-val $> 0.154$)

Global assortativity coefficients (*r*) of individual sNETs and fNETs, as reported in the literature, were calculated to serve as a baseline, for all 3 stages of clinical cognitive decline. Figure 2 depicts the global assortativity distributions for each group. In agreement with the literature, sNETs are assortative ($$r>0$$), while fNETs’ global assortativity coefficient is close to 0 and primarily shows disassortativity ($$r<0$$). This dichotomy between sNETs and fNETs corroborates the results of previous research [38]. Despite this consistent difference between the global assortativity coefficients of sNETs and fNETs, neither one shows significant variability between the stages of clinical cognitive decline (p-val $$ > 0.154$$).

**Fig. 3:**
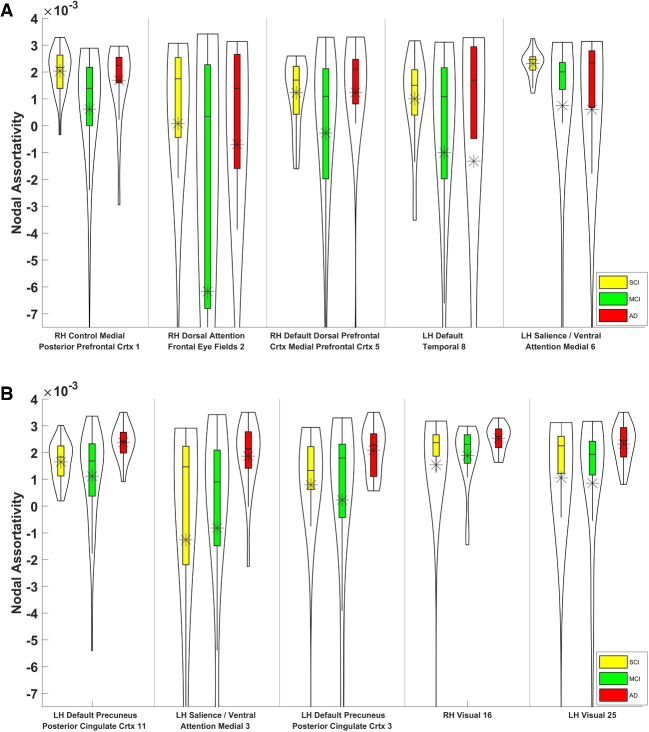
Distributions of nodal assortativity coefficients of the top 5 most discriminative sNET nodes are shown for SCI-vs-MCI (p-val $< 0.018$) at the top panel and for MCI-vs-ADD (p-val $< 0.004$) at the bottom panel. A statistically significant decrease in nodal assortativities is observed in the transition from SCI to MCI, while statistically significant increase is observed in transition from MCI to ADD, albeit at mutually exclusive sets of nodes (cortical regions)

Statistical assessment of the discriminative power of nodal assortativities, as described in Section “Identification andverification of relevant local changes” revealed right control medial posterior prefrontal cortex (1), right dorsal attention frontal eye fields (2), right default dorsal prefrontal cortex medial prefrontal cortex (5), left default temporal (8), left salience / ventral attention medial (6), as the top 5 significant sNET nodes, right control lateral prefrontal cortex (10), right somatomotor (13), left visual (21), right default precuneus posterior cingulate cortex (3), right default precuneus posterior cingulate cortex (1), as the top 5 significant fNET nodes for SCI-vs-MCI classification. The corresponding top 5 significant sNET and fNET nodes for MCI-vs-ADD classification are left default precuneus posterior cingulate cortex (11), salience / ventral attention medial (3), left default precuneus posterior cingulate cortex (3), right visual (16), left visual (25) and left somatomotor (22), right default dorsal prefrontal cortex medial prefrontal cortex (9), left limbic temporal pole (8), left control parietal (5), left somatomotor (5), respectively.

**Fig. 4:**
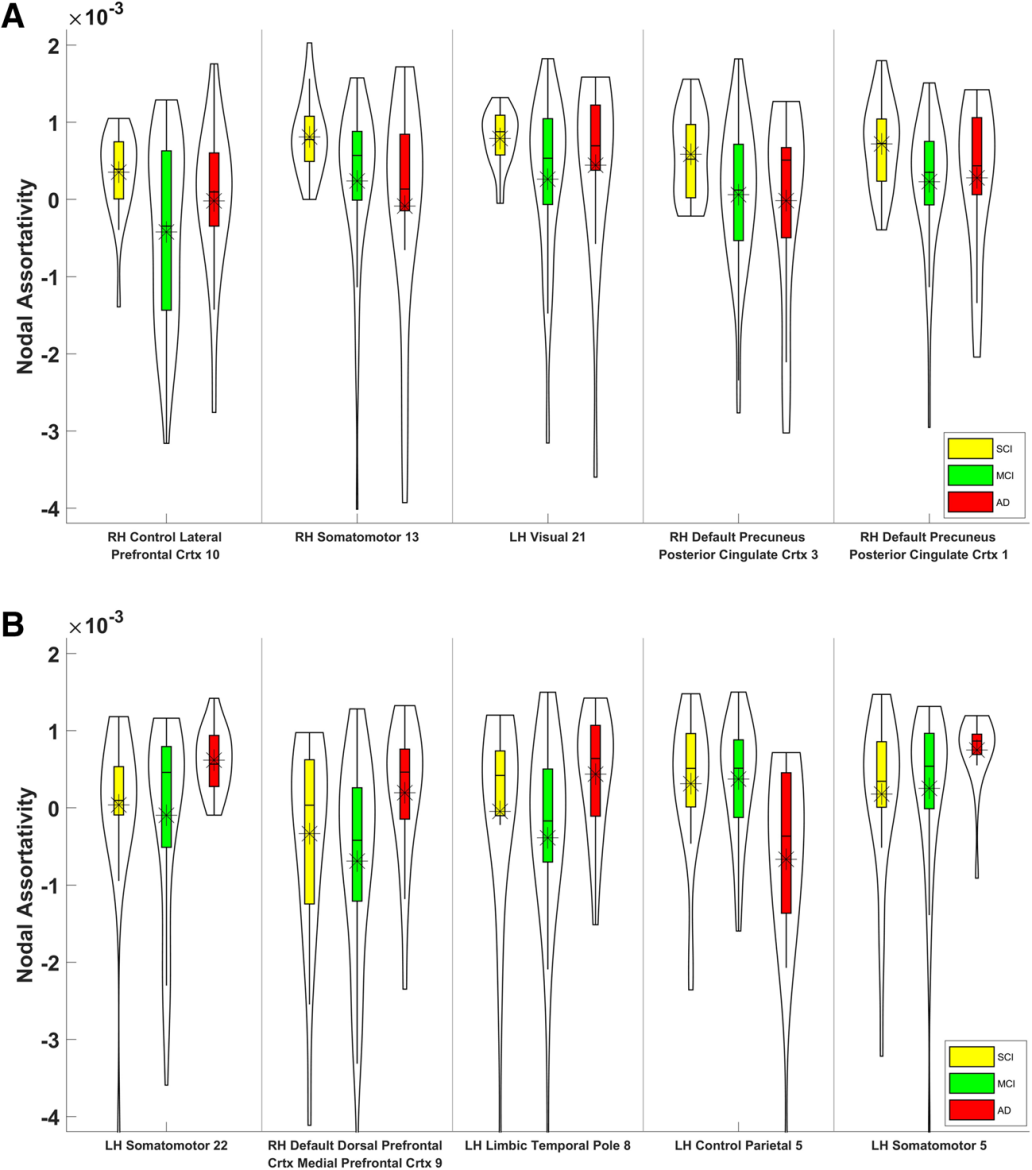
Distributions of nodal assortativity coefficients of the top 5 most discriminative fNET nodes are shown for SCI-vs-MCI (p-val $< 0.002$) at the top panel and for MCI-vs-ADD (p-val $< 0.012$) at the bottom panel. A statistically significant decrease in nodal assortativities is observed in the transition from SCI to MCI at all 5 nodes, while a statistically significant increase is observed in the transition from MCI to ADD at 4 of 5 nodes and a decrease is observed parietal (5) within control network in the left hemisphere. The identified node sets (cortical regions) for both transitions are mutually exclusive

Figure 3 reports the distribution of the nodal assortativity coefficients of selected sNET nodes across all 3 groups. Figure 3A reveals a significant reduction in the nodal assortativity as the severity of cognitive decline increases from SCI to MCI (p-val $< 0.018$), for all SNET nodes that are in top 5 in terms of their statistical significance for SCI-MCI classification. Conversely, the top 5 SNET nodes with the highest discriminative power for MCI-vs-ADD classification show an increase from MCI to ADD (p-val $< 0.004$), as shown in Fig. 3B. It is noteworthy that these two sets of nodes are mutually exclusive.

Figure 4 reports the identical statistical analysis of nodal assortativity for fNETs. Similarly to sNETs, data in Fig. 4A reveals a statistically significant decrease in nodal assortativity of selected fNET nodes as the severity of cognitive decline increases from SCI to MCI (p-val $< 0.002$). As the disease progresses from MCI to ADD, we find that 4 of the top 5 fNET nodes again display an increasing nodal assortativity (p-val $< 0.012$) while one node (Control Parietal 5 in the left hemisphere) has the opposite trend, as shown in Fig. 4B. These two sets of nodes are also mutually exclusive.

In order to investigate the potential influence of age as a covariant on our findings, we computed the Pearson’s correlation coefficient between age (as an independent variable) and each of the five identified nodal assortativities (as dependent variables) and also performed linear regression analysis. The absolute values of the Pearson’s correlation coefficients ranged from 0.01 to 0.30. The significance of the linear regression models was assessed using $R^2$ statistics and F-test p-values, with ranges of [0.0001, 0.04] and [0.08, 0.93], respectively. These results indicate that the predictive power of the five nodal assortativity measurements does not show a statistically significant correlation with age.

Overall, we observe a consistent and statistically significant decrease in nodal assortativity in specific brain regions as clinical cognitive decline progresses from SCI to MCI, and a significant increase in nodal assortativity from MCI to ADD. This is observed in both sNETs and fNETs (except in one fNET node), albeit at mutually exclusive locations for sNETs and fNETs, as well as for the SCI-to-MCI and MCI-to-ADD stages.

### Relevance of identified nodes as potential diagnostic biomarkers

In order to verify the relevance of the selected nodes as potential low dimensional (hence robust) diagnostic biomarkers, we trained linear SVM classifiers^5^ and tested them separately for each classification task (SCI-vs-MCI and MCI-vs-ADD) and each connectome type (structural and functional) by using leave-one-out experiments. Since the majority of prior work focuses on SCI-vs-ADD classification, we report the classification performance on this task as well for comparison. We further conducted experiments using the full-connectome^6^ (leading to $400\text{- }D$ feature vectors) which is also a common approach reported in literature [19]. Finally, all experiments were repeated by replacing the nodal assortativity with alternative, popular nodal features in the field of complex networks, namely, clustering coefficient (CC), betweenness centrality (BC), node strength (NS) and node degree (ND) for comparison.

Table 2 lists the accuracy values for all the experiments conducted. The results indicate that the 5*D* feature vectors derived from select subset of nodes are consistently superior to high-dimensional, whole network representations, irrespective of the particular network measure chosen. It is also observed that, on average, using nodal assortativity performs better than using other local network characteristics in SCI-MCI-ADD classification. Employing the nodal assortativity of the selected nodes yielded an accuracy value in the range of $0.75 - 0.90$ in all three classification tasks and for both types of connectome.

The results of the multiple random shuffling experiments, as described in Section “Identification and verification of rel-evant local changes” and also given in Table 2, offer further evidence for the relevance and statistical significance of the identified nodes as regions affected during different stages of clinical cognitive decline. Such a test is also necessary to address a possible criticism of the use of the available limited dataset simultaneously for feature selection and classification. We find that random reassigning SCI/MCI/ADD labels to the study group and repeating the analysis from the beginning to the end result in a significant drop in accuracy for all classification tasks when compared to the non-shuffled (true-label) case. The use of statistically most significant nodes for classification on the same data yields a typical accuracy of 50-60% on randomly shuffled labels, which we take as a baseline. In comparison, the classification accuracy observed with the true labels ranges from 75% to 90%. We therefore conclude that the high diagnostic power of the top-scoring cortical regions is not a generic outcome of the method we employ here, but is indicative of the clinical relevance of the identified cortical regions as sites where relevant local topological changes in connectivity occur. The observation that changes in these regions (connectome nodes) can be used to predict the corresponding stage of cognitive decline is further corroborated by the fact that the direction of change in nodal assortativity is practically unanimous within each node set (also a feature that is lost under random shuffling of class labels).

**Fig. 5:**
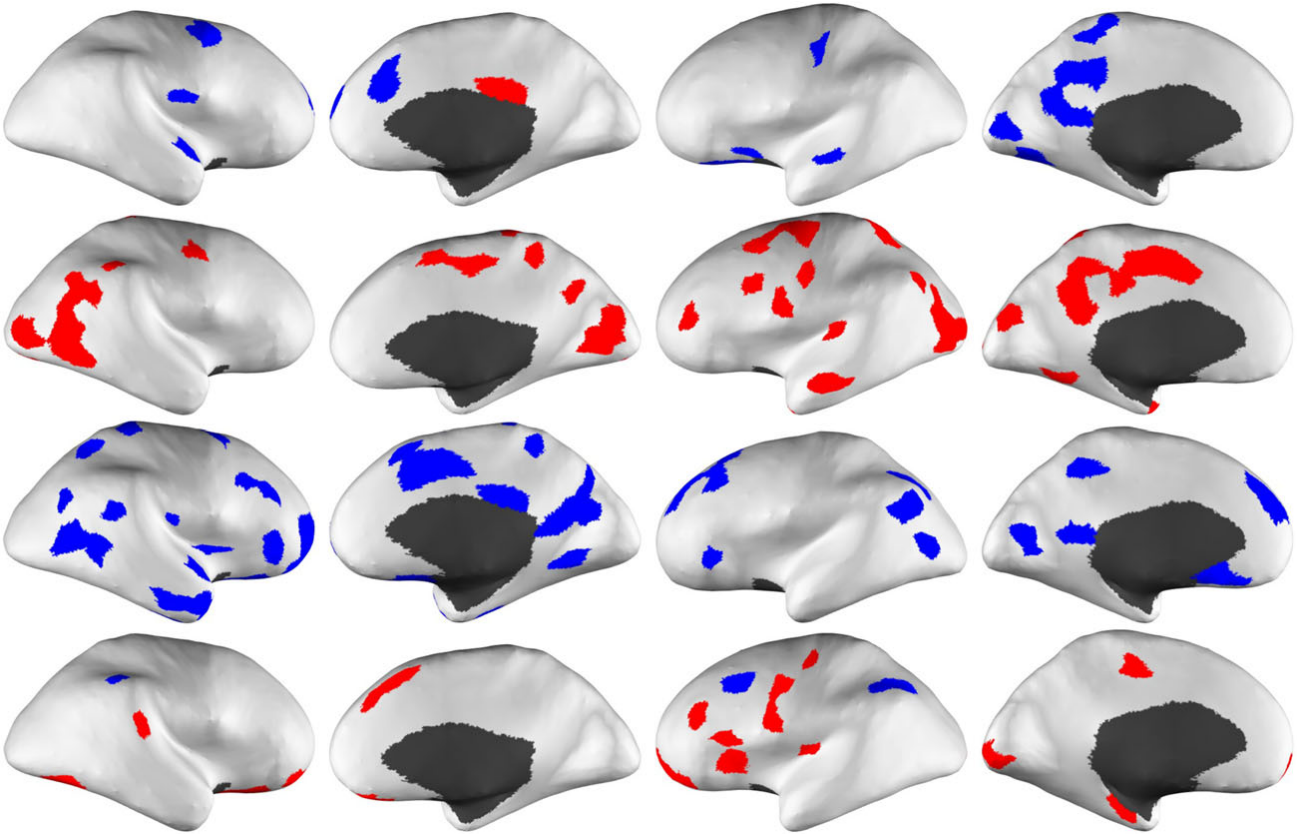
Differential nodal assortativity maps of the statistically significant sNET locations for SCI-vs-MCI **(1st row)**, MCI-vs-ADD **(2nd row)** discrimination and fNET locations for SCI-vs-MCI **(3rd row)**, MCI-vs-ADD **(4th row)** discrimination, displayed from lateral/medial views of right/left hemispheres, respectively, on inflated brain surface. (BrainSurfer [39]). The cortical regions with a statistically significant increase/decrease in assortativity with increasing cognitive decline are depicted in red/blue, respectively. In general, a nodal assortativity decrease, suggesting an increased vulnerability, is observed in the first half of cognitive decline, while an increase in nodal assortativity indicating increased local connectivity supporting resilience, is observed in the second half. Spatial variability is also clearly observed

We trained linear SVM classifiers by including age as the sixth feature to further assess its impact as a covariant. The comparison of the classification results with and without age, using a paired-sample t-test, did not show any statistically significant difference (all p-values $>0.60$ across all classification tasks). The feature importance weight assigned to age by SVM is also smaller by an order of magnitude than the second least significant feature. These results also confirm the statistical significance of the identified nodal assortativities across the clinical cognitive decline spectrum as a biomarker.

## Discussion

The global assortativities of sNETs and fNETs show a significant difference, irrespective of the clinical cognitive decline stage, with the former demonstrating higher global assortativity than the latter. This observation is in agreement with the literature and confirms that global assortativity of neither sNETs nor fNETs can be used as a diagnostic biomarker. However, the proposed local (nodal) assortativity analysis revealed statistically significant differences between different stages of clinical cognitive decline, albeit at varying locations and nature for structure and function.

The most significant sNET nodal assortativity changes in the first half of the clinical cognitive decline spectrum, namely from SCI to MCI states, are observed most conspicuously in the left precuneus posterior cingulate cortex (pCunPCC) in DMN, the left orbital frontal (Limbic), the right medial posterior prefrontal cortex (control network - CON), the right frontal eye fields (Dorsal Attention network - DAN), the left posterior (DAN) and in the right cingulate (CON) with a significant decrease in nodal assortativity except the last one. Ide et al. pointed out that nodes with a high relative vulnerability tend to exhibit disassortativity (decreased assortativity) [40]. Murakami et al., on the other hand, had associated decreased local assortativity with shorter (and faster) local communication paths [35]. Hence, our observations suggest that certain nodes in sNETs take over increased roles in communication, resulting in greater local vulnerability. In contrast, the most significant sNET nodal assortativity changes from MCI to ADD manifest themselves in the form of local assortativity increase. The most conspicuous regions where such an increase is observed are the left pCunPCC and right parietal (DMN), the left lateral prefrontal (CON), the left temporal pole (Limbic), the somatomotor cortices (SMN), the midcingulate cortex (salience-ventral attention network - SVN) in the right and left hemispheres, the right lingual gyrus & cuneus, the occipitotemporal junction (visual network - VN) and the left occipital pole (VN) (Fig. 5).

These observations can be interpreted as a compensatory response to connectivity disruptions observed in MCI, in the form of alternative connections among brain regions. The increase in nodal assortativity in the temporal and frontal regions can be linked to a decrease in nodal efficiency which indicates a reduction in the efficiency of information flow, predominantly identified in the frontal and temporal lobes, as reported in literature [16]. Jones et al. suggested that the initial disruption of the posterior DMN could eventually lead to enhanced connections between the posterior DMN and other central hubs in the brain [41]. This is partially supported by our results which indicate spatially different regions undergoing local changes of different nature across the spectrum. The importance and effectiveness of tracking local changes are also supported by the high diagnostic power of the selected nodal assortativities.

Similar changes are observed in fNETs, albeit mostly in different locations than the ones in sNETs. A significant decrease in fNET nodal assortativity from SCI to MCI was most conspicuous in the bilateral pCunPCC (DMN), the left prefrontal cortex (DMN), the bilateral parietal (DMN), the right lateral prefrontal cortex (CON), the right cingulate (CON), the left & right orbital frontal (limbic), the right temporal pole (limbic), the right MCC (SVN), the right posterior (DAN), the somatomotor cortices, the right occipitotemporal junction (VN) and the left cuneus (VN). It should be noted that fNETs are models of correlative function. Hence, this decrease in nodal fNET assortativity, which is a wider spread phenomenon compared to that of sNETs in the first half of the spectrum, is not necessarily indicative of an increase in local vulnerability but, rather, may be the result of a functional re-organization of the brain where certain nodes start orchestrating function. A possible explanation, in parallel with the explanation suggested for the local assortativity decreases from SCI to MCI, is that few new subnetworks may have been built. These findings are also in agreement with the loss of functional connectivity in DMN and in the bilateral orbital frontal cortex within the limbic network [42, 43]. The nodal assortativity increase from MCI to ADD, on the other hand, is spatially more limited. This increase is most conspicuous in the right dorsal prefrontal cortex medial prefrontal cortex (DMN), the left prefrontal cortex (DMN), the right & left orbital frontal (limbic), the left temporal pole (limbic), and the right & left lateral prefrontal cortex (PFCl) (CON), the left frontal operculum insula (SVN) and the lateral & somatomotor cortices, accompanied by a nodal assortativity decrease in the left parietal (CON). The increase in nodal assortativity within the temporal pole can potentially be due to increased functional connectivity in this region reported for ADD [44]. The observed increase in assortativity in the prefrontal cortex, which is in agreement with prior findings reported in literature, suggests that ADD may be making an effort to enhance prefrontal connectivity in order to sustain cognitive efficiency in the face of limited DMN resources [42] (Fig. 5).

Irrespective of the underlying topological changes in brain connectome (network) organization, it is clear that the brain’s structural topology and functional organization undergo local changes in spatially segregated regions across the clinical cognitive decline spectrum. The change is consistent with the stage of the cognitive decline, but spatially different in different stages and (to a great extent) between structure and function. The observed spatial differences in sNETs and fNETs should be interpreted with caution as these two types of connectomes represent different aspects of brain organization. While the observations in sNETs can be attributed to local topology and communication efficiency, the observations in fNETs are more likely to be associated with functional organization, eg. in terms of functional units.

A comparison of local and global network measures, as summarized in Table 2, shows that local measures have a higher diagnostic power than global measures. Among all local measures, the local (nodal) assortativity is the single measure with a strong discriminative power across the clinical cognitive decline spectrum. Hence, although other measures can capture certain changes in certain stages of cognitive decline, nodal assortativity singles out as a potentially powerful measure for not only diagnosis but also for disease progress monitoring.

## Conclusion

Clinical cognitive decline, leading to Alzheimer’s Disease Dementia (ADD), has long been considered as a disconnection syndrome manifesting itself in the structural and functional brain organization. Despite the evidence in literature with regard to significant changes in (primarily functional) brain connectome models, these efforts have not yet provided a coherent insight into underlying mechanisms, nor offered strong biomarkers. We investigated changes in local structural and functional reorganization of brain at different stages of clinical cognitive decline, using local (nodal) assortativity as a quantitative measure. We observed statistically significant local reorganization of brain that not only vary with the progress of the cognitive condition but also differ in nature between structure and function that manifested in the form of spatially varying hyper and hypo-assortativity in sNETs and fNETs. More specifically, hypo-assortativity is generally observed in the first phases for both function and structure and hyper-assortativity for later stages. The reorganization is more prominent in function in early stages and in structure at later stages. The affected regions for structural and functional organization do not overlap, though some common locations were observed. It is imperative to emphasize the significance of analyzing both spatial and temporal dimensions to achieve a comprehensive understanding of the clinical cognitive decline leading to Alzheimer’s Disease. Our preliminary findings suggest a complex and variable (non-monotonous) reorganization that may hint simultaneously active degenerative and compensatory mechanisms. Further research from such a multi-dimensional perspective on a larger dataset, and ideally with follow-up data, has the potential to provide an alternative picture of the brain’s reorganization during the course of cognitive decline.

## Data Availability

The data are not publicly available due to because data sharing requires the funding agecy’s (TUBITAK) approval. The data presented in this study are available on request from Burak Acar.

## Footnotes

1 https://surfer.nmr.mgh.harvard.edu/

2 Istanbul University, Istanbul Faculty of Medicine, Ethics Committee Approval: 877/30.05.2014; Bogazici University, Ethics Committee Approval: 2014-1/17.02.2014

3 The preprocessing and co-registration pipeline is available at https://vavlab.boun.edu.tr/brainet-structural-and-functional-brain-network-analysis

4 The *DiffusionTensor3DReconstructionImageFilter* from ITK v6.0.0 is used.

5 MATLAB R2023a™, *fitcsvm* with linear kernel, automatic kernel scaling is used with prior class probability set to *uniform* to compensate for imbalanced training dataset.

6 The experiments with a $400\text{- }D$ feature vector are denoted with a (*) in Table 2
